# Patient perceptions concerning clinical trials in oncology patients

**DOI:** 10.1016/j.conctc.2016.09.005

**Published:** 2016-09-21

**Authors:** A.L. Dias, J.H. Chao, D. Lee, Y. Wu, G.H. Kloecker

**Affiliations:** Department of Medicine, Division of Hematology and Medical Oncology, James Graham Brown Cancer Center, 529 S. Jackson Street, Louisville, KY 40202, USA

**Keywords:** Clinical trials, Phase 1 trials, Randomized trials, Investigator initiated trials

## Abstract

**Background:**

Clinical trials are critical to scientifically evaluate promising new therapies in oncology, but patient accrual to these studies is persistently low. Patient preference plays an important role in enrollment in these trials. We performed this survey to evaluate the perceptions of newly diagnosed oncology patients about clinical trials and the reasons why they wish to or not to participate in these trials.

**Methods:**

Patients were given a ten question survey reflective of their attitudes regarding clinical trials as a treatment option at their initial visit. The self-directed questionnaire was scored on an ordinate scale from strongly agree [1] to strongly disagree [5].

**Results:**

Ninety three patients were surveyed in the cancer specific multispecialty clinics in an academic center. Our patients expected their providers to discuss all information relating to clinical trials and eligibility at the first visit (65.4% agree and 15.4% neutral, p < 0.0001). Patients felt their privacy and safety would be safeguarded in the University sponsored trials (56.8% agree, and 25.7% neutral, p < 0.0001). Over 80% patients showed their unwillingness to participate in randomized clinical trials (disagree 61%, neutral 19.5%, p < 0.001). Patients also showed less likelihood to participate in clinical trials as a first treatment option (48.7% disagree and 28.9% neutral, p0.0161), but were willing to consider participating in a clinical trial if the conventional treatment failed. Industry sponsored trials, phase 1 trials, investigator initiated trials with the involved tests and time commitment and altruistic reasons did not significantly deviate from the mean preference analyzed using Fisher's exact test analysis.

**Conclusions:**

Patients consider the option of clinical trials as important in their treatment, and expect to be informed by their oncologist about such trials. Newly diagnosed cancer patients perceive randomization and first line trials negatively. Since randomization data provides new standards of care and hope for improved treatment, patients and their families must be educated of their importance.

## 1 Introduction

The efficacy of new cancer treatments can only be reasonably confirmed through carefully designed prospective clinical trials. A key requirement in the conduct of these trials is the enrollment of sufficient numbers of participants to establish adequate statistical power and make it applicable to the intended population. In addition, clinical trials offer cancer patients access to innovative therapeutic approaches and have the potential of improving clinical outcomes. A meta-analysis done in the past has shown that centralized referral or entry into clinical trials was frequently associated with higher survival rate [1]. However only a few eligible cancer patients are recruited to clinical trials [2], [3], [4]. This obvious threat to timely completion leads to delays and decreased access to effective treatment plans.

It has been estimated that less than 5% of cancer patients worldwide are enrolled in clinical trials [5]. In addition only approximately half of these clinical trials reach the designated minimum accrual numbers [6]. A recently published review from Australia recognizes that only 2–3% of adult cancer patients participate in clinical trials compared with 50% of pediatric patients [7]. This review critically analyses three main obstacles-clinician, patient and system. Clinician behavior is the most important of these with the observational studies suggesting clinicians may not be offering participation to a large proportion of patients they know to be eligible [7]. Lack of awareness of trials is the other common obstacles to accrual [8], [9], [10]. Communication between clinician and patient appears to be a greater issue than previously recognized. The most contentious issue is still the potential benefit of participation to an individual patient. There have been three recent systematic reviews of this topic that reached different conclusions as to the evidence of benefit [1], [11], [12]. Arguably, the most complete of these found that evidence of benefit was greater in childhood cancer, hematological malignancies and for trials conducted before 1986 [11].

We conducted this trial with a primary objective to establish patient's perspectives on participating in clinical trials. Initially we discussed the importance of clinical trials in developing new drugs for treatment of cancers, and cancer related events and their understanding of what it means to them to participate in the trials along with their expectations from the trials. Our survey constitutes a better understanding with regards to beliefs and myths of participating in trials. We hope these findings will provide more insight to the oncology practitioners to optimally devise a strategy to improve patient recruitment to improve accrual rates in future trials.

## 2 Methods

A single institution cross sectional study design was employed, using a set of questionnaires developed to capture the trials related attitudes and opinions of patients. The study was performed at the James Graham Brown Cancer Center, part of the University of Louisville Hospitals in Kentucky. The study had the institutional review board approval and was performed from March 2012 over a one year period. Questionnaires were developed for patients to examine their attitudes and beliefs regarding the various issues surrounding the process of trial recruitment. First a literature search was performed to identify factors previously reported to influence patients' decision of participating in clinical trials.

### 2.1 Data collection

We surveyed cancer patients diagnosed with all non-hematological malignancies for one year beginning March 2012. The eligibility criteria included [1], age 18 years or older [2], histological confirmation of a solid tumor [3], life expectancy over 2 months and [4] an Eastern Cooperative oncology group (ECOG) performance status of 0–2. . Patients who already had initiated treatment were excluded. Physician consent was obtained prior to patient enrollment. Written consent to participate in the study was then obtained from the patients.

One well trained research nurse explained the purpose of the survey and received written informed consent from all participants before they were enrolled in the study. With the agreement of the participants, clinical information of each patient was collected from the medical records as and when needed. The study design and survey instruments were approved by the Institutional Review Board, University of Louisville Hospital.

### 2.2 Questionnaire

Patients reported their age, gender, level of education, ethnicity and living status. The type of cancer and the stage was recorded by the interviewer. Education was recorded as the highest grade obtained at the time of diagnosis. All information was kept confidential.

The standardized self –administered questionnaire consisted of 15 questions developed in collaboration with the biostatistics department and the clinical trial division at James Graham Brown Cancer center. Five questions concerned demographics that included age, sex, ethnicity, gender and educational level. The remaining 10 questions asked about the cancer clinical trial understanding and their perception. A score between 0 and 5 was used in the survey to evaluate each of the 10 questions. A score of 1 indicated “agree strongly” and 5 indicated “disagree strongly”. The questionnaire format is described in the attached addendum (addendum 1).

### 2.3 Statistical methods

All data generated was centrally reviewed and analyzed using the Fisher's exact test to examine whether the response variable is associated with each covariate, such as gender, age, cancer type, ethnicity and education level and the p values obtained. A biostatistician was consulted for all statistical designs and analyses and all tests were considered statistically significant at a p < 0.05.

## 3 Results

From March 1, 2012 to April 30 2013, a total of 93 patients were asked to participate in the study, and 85 patients (93.41%) agreed to complete the questionnaire. Four patients declined to participate and four returned the questionnaire incomplete. The median age of the cohort was 60 years with a range of 29–84 years. The group was matched for sex distribution with 45 males (51.14%) and 43 (48.86%) females. 81% patients interviewed were Caucasians. Over 90% of the enrolled patients were educated to higher than 8th grade, with 40% reporting college or postgraduate qualification. 37 (41.6%) were involved in decisions for lung cancer trials, 20 (22.5%) in melanoma cancer trials, 14 (15.7%) in breast cancer trials and remainder in colorectal, pancreatic and other cancers.

Over 80% patients wanted their treating physician to inform them about the ongoing clinical trials and their eligibility (51, (65.4%) agree and 12 (15.4%) were neutral), with only 15 (19%) not wanting to know of the trials, making this statistically significant (p < 0.0001). There was no difference based on the gender, level of education or type of cancer regarding this although only 50% of African Americans were interested to know of the trials (6 of the 12 patients). 83% of the patients (42 (56.8% agree, 19, (25.7% neutral) agreed that the University sponsored clinical trials were protective of their privacy and identity. This also reflected in the opinion on safety where 78% of the patients (38 agreed and 19 were neutral) opined to be safe in a trial sponsored by the University. Chi-square test done to test independence of safety and privacy maintenance of trials conducted by the University showed no statistical relationship.

On the other hand patients indicated a strong level of disagreement of participating in randomized trials (“if enrolled in a clinical trial, I am comfortable being assigned by a method such as flipping a coin or throwing a dice) with 80% (47 (61%) disagree and 15(19.5%) neutral) unwilling to participate in randomized trials, and this was statistically significant (p < 0.0001). In addition significant number of patients did not agree to consider participating in phase 1 trials as a first treatment option (37 (48.7%) disagree and 22 (28.9%) neutral). However over 67% of patients (33 (42.9%) agree and 23 (29.9%) neutral) were willing to consider participating in a clinical trial if the conventional treatment failed.

**Fig. 1:**
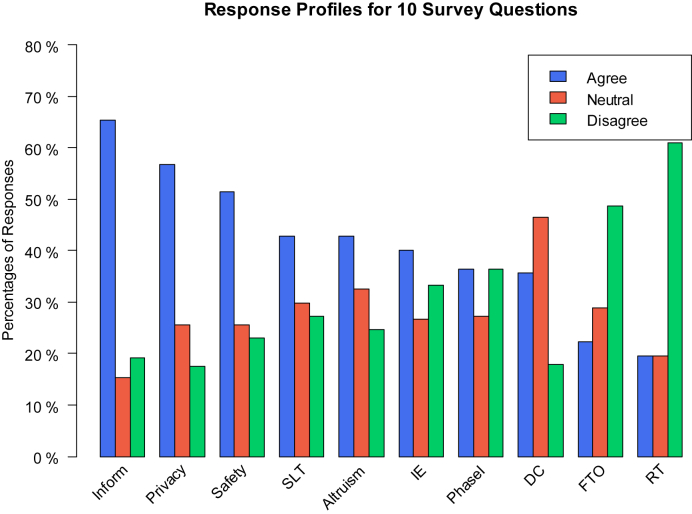
Response Profiles for 10 Survey Questions. Inform (Q3), Privacy (Q9), Safety (Q10), SLT (Second Line Treatment, Q6), Altruism (Q7), IE (Increased Effort, Q8), Phase I (Q2), DC (Drug Companies, Q1), FTO (First Treatment Option, Q5), RT (Randomized Trial, Q4). The responses of first three questions are significant different from the last three (Inform & RT: p < 0.0001, Privacy & FTO: p < 0.0001, Safety & DC: p = 0.0299).

Other factors which influenced decision making in participating in clinical trials were if the trials were sponsored by drug companies. In this regard, where majority of the patients were neutral or undecided, 34 (46.6%) and 13 (17.8%), not willing to participate in drug company sponsored trials. Mixed responses were obtained with benefits and risks involved with the phase 1 drugs when questioned - “I am less likely to participate in a clinical trial if I might receive a promising new drug even if its positive and negative effects are uncertain”. Equal number 28 (36.4%) patients agreed or disagreed for participation with another 21 (27.3%) were neutral or undecided. Finally with regards to altruistic behavior pattern significant number of patients agreed or remained neutral 33 (42.9%) and 25 (32.5%) as against 19 (24.7%) who disagreed. Most patients did not mind the time and additional tests required with the clinical trials, 30 (40%) agreed and 20 (26.7%) were neutral versus 25 (33%) who were not willing to spend time and do additional tests as required by the trials (Appendix 2 and Fig. 1).

## 4 Discussion

Our study is an attempt to identify the opinions and attitudes of patients and their expectations about the benefits of participating in clinical trials. In particular we examined patients perceptions with regards to information relating to the available trials, drug company sponsored trials as against University sponsored trials, trial confidentiality and safety issues, chance of benefit from the experimental agent to self or to others, and their overall perception of clinical trials.

The predictors identified in this study are not unique, but they do present possible targets for intervention for increased patient accrual in clinical trials. Overwhelming majority of our patients wanted information on the prevailing clinical trials and their eligibility. We know that effective patient-physician communication is critical for patient accrual in clinical trials. Past studies have shown that patients want a high degree of information and the information usually produces a positive effect [13]. Recent study has shown that the attitude of patients is generally in favor of participation in clinical trials and good communication with the treating physician plays a dominant role in decision making [14]. Past surveys have also shown physicians underestimate patients' ability to comprehend and desire to obtain information [14]. It is also known that physicians have difficulties in estimating the amount and type of information that patients want and their effectiveness in imparting information [15]. It then becomes the primary responsibility of the physician to impart factual, reasonable and appropriate information so that the patient is appropriately informed and enabled to make reasonable decisions relative to treatment options.

Recent studies have shown there is an overall increased awareness regarding clinical trials between 2008 and 2012, although large section of population still lacks general awareness [16]. Racial and ethnic disparities in trial awareness exist although disparities may be decreasing among the minorities [16]. However despite this awareness there appears a reluctance to participate in a clinical trial as the first treatment option as evidenced in our study. There are many reasons for this perception. Studies in the past have shown that some patients thought that they would be used as “guinea pigs”, that proposed participation in a clinical trial was for the sole economic interest of the physician and that physicians propose clinical trials even if there are other drugs that could be more effective than the drug used in clinical trial [17]. Although patients have these concerns they are not discussed during the conversation with the doctor, who is concentrated on explaining the proposed study; furthermore they are not usually cited in the informed consent and patients do not request discussion of these concerns [18], [19]. This could have the consequence that patients, not having discussed and clarified these issues, could be influenced by them when making their decision whether or not to participate in the proposed study, without being able to decide on the objective reality of the study itself and in the final analysis, without being able to make either a free or an informed decision [20]. We need to remember several studies in the past have shown patients perception of personal benefit to be the best predictor of clinical trial entry [21]. We strongly recommend patients should have an open discussion about the benefits as well as risks of then participating in trials and all concerns expressed by them to be duly addressed. We need to highlight here that majority of our patients were willing to consider participating in clinical trials if the conventional therapies fail and/or if there are no other suitable alternatives.

Randomization is one of the most common protocol related barriers to participation [22], [23]. Our study confirmed this finding wherein more than 80% of our patients were not keen to participate in randomized controlled trials. Researchers in the past have tried to deduce reasons for poor accrual and perhaps random allocation of treatment is a major reason why patients chose not to join randomized clinical trials [24], [25]. Other reasons include a poor understanding of the rationale for randomization as a method of treatment allocation [25], [26], and difficulty to comprehend the complex terms used such as blinding or placebo [22]. Studies also suggest, patients want to be actively involved in clinical decision making and they prefer either the doctor or themselves to make the decision about which treatment they will receive [27], [28]. Other factors reported in the literature to be influential in patients' decision to participate in randomized clinical trials include objection to be an experimental subject, or report feeling like a “guinea pig” [29], distrust of the medical profession and a lack of knowledge of what is required of trial participants [30]. Trials in which there are large differences in the treatments offered, particularly in regard to toxicity or the possibility of receiving a placebo often experience greater recruiting difficulties [31]. Randomization continues to be a concept that influences participation in research studies and we suggest this should be a barrier addressed in meetings with patients. Patients may feel more comfortable and more open to enrollment with an in-depth and a clearly explained description of randomization.

Our survey also showed, patients were highly concerned with maintenance of privacy and safety involved with the trials. They felt more assured and safe with the University sponsored trials. However there were nearly a quarter of the patients who were skeptical of their safety and privacy even with University funded clinical trials. Past studies have confirmed patient fears of safety, side effects, efficacy of treatment offered and fear of placebos [32]. Studies have reported treatment severity and worry about efficacy were the top rated fears [32]. In the recent years there has been a dramatic fall in public trust in medical establishments and drug companies worldwide, since unethical behavior by some violators has become visible [33]. Trust is an important factor that affects how individuals view clinical trials and it contributes to whether they ultimately are willing to participate in clinical trials [34], [35], [36], [37]. To counteract distrust that is often felt by patients information on protection of research subjects should be widely disseminated to the public and doctors should clarify to patients the laws formulated to protect them.

In the past it has been argued that only reason patients would participate in clinical trials is that they choose to be altruistic. Trials have shown that benefitting others and advancing medical knowledge were highly rated as reasons for participating in clinical trials [38]. Patients in our study were no different with half the participants willing to participate in clinical trials if it helped other patients and to advance medical progress.

Finally, in our survey industry sponsored clinical trials were not viewed favorably when compared to the University sponsored trials. It is difficult to determine the reasons. Past studies and surveys have explained several reasons for negative perceptions with regard to industry conducted trials, the most dominant perception among physicians and patients being that they skew the research they sponsor to make their drugs look better and safer [39]. We believe the major concern for this poor understanding is lack of knowledge of trials conducted by pharmaceutical companies. Also our surveys patients felt privacy and safety would be better safeguarded by the University conducted clinical trials. Additionally despite having to undergo frequents clinical and laboratory visits, nearly half of the surveyed patients were willing to spend extra time and do the additional tests required by the clinical trials.

Our study is not without limitations. Our sample size is small. As a result we are not able to statistically segregate differences based on gender, race, socioeconomic status, cancer type, rural vs. urban population, religious beliefs, and the educational level. Although we did plan a large trial, recruitment for this survey was faced with difficulties. We surveyed newly diagnosed cancer patients and hence there would have been scope for very high anxiety. We used an unvalidated questionnaire in our survey and hence may have been a confounding factor in our analysis on perceptions of randomized trials and industry run trials. These factors were identified in literature as factors of concern when recruiting patients for clinical trials.

## 5 Conclusion

The common goal of the growing interest in clinical trials related research is to increase patient accrual and accelerate the pace of clinical research. The results of this small study suggest perhaps that a simple straightforward solution to increase patient accrual is not forthcoming. Our study stresses on the importance of effective communication between physicians and patients not only to explain study design and the benefits, but also to improve accrual rates. There are a number of areas that warrant further research and specifically there is a need to raise community awareness about the need for and importance of randomized clinical trials. The timing of such education also should be the focus of research. Efforts to improve our understanding of those factors involved and the development of interventions to improve informed consent and support patients in their decision making should continue.

## Figures and Tables

**Figure:**
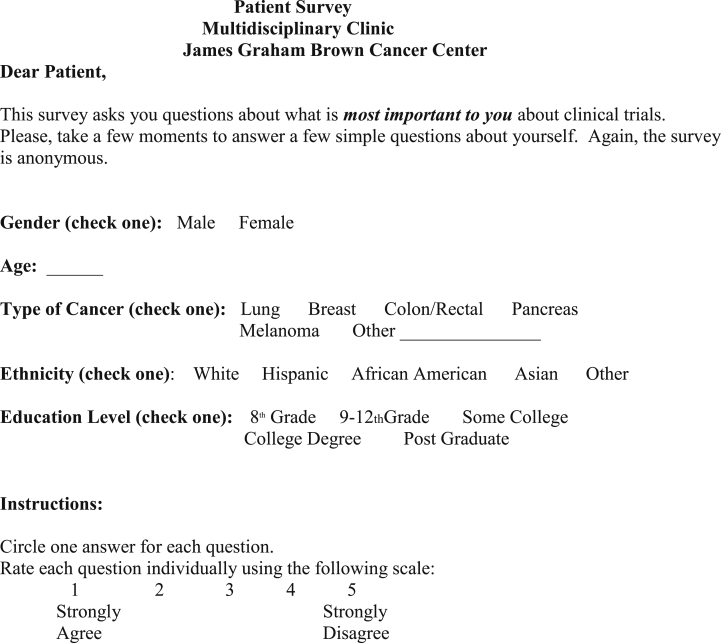

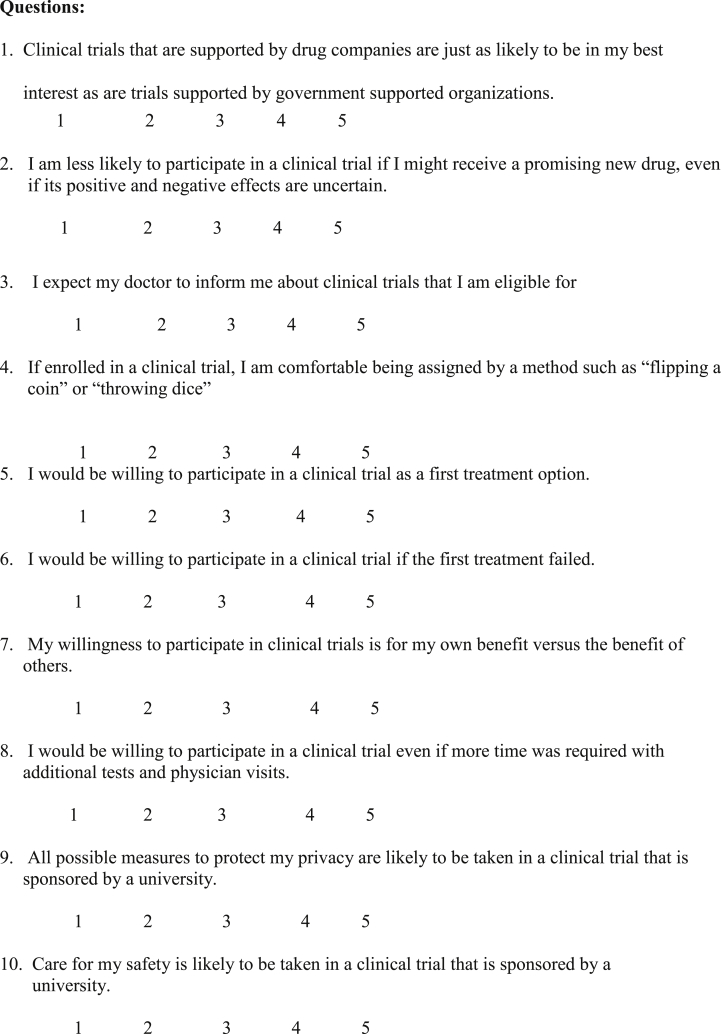


**Table:** Patient Characteristics

Characteristic		Frequency (n = 89)	%
Age	Median (years)	60 ± 10	
	≤60	46	51.7
	>60	43	48.3
Sex	Male	46	51.7
	Female	43	48.3
Race	Caucasians	72	81.82
	African Americans	16	18.18
Education	Post graduate	5	5.68
	College/Degree	30	34.09
	High school	47	53.41
	Middle school (grade 8)	4	4.55
	Not available	2	2.27
Type of Cancer	Lung	37	41.6
	Melanoma	20	22.52
	Breast	14	15.7
	Colon/Rectum	6	6.7
	Pancreas/others	12	13.5

**Table:** 

	Total	Agree (%)	Neutral (%)	Disagree (%)	p-value
1. Drug Company trials vs government supported trials	73	26 (35.6)	34 (46.6)	34 (46.6)	0.0071
2. Promising new drug not knowing effect (phase 1)	77	28 (36.4)	21 (27.3)	28 (36.4)	0.5176
3. Information on clinical trials	78	51 (65.4)	12(15.4)	15(19.2)	<0.0001
4. Randomized trials-“Flipping a coin” or “throwing a dice”	77	15 (19.5)	15 (19.5)	47 (61)	<0.0001
5. Clinical trial as first line option	76	17 (22.4)	22 (28.9)	37 (48.7)	0.0161
6. Clinical trial after first line treatment failed	77	33 (42.9)	23 (29.9)	21 (27.3)	0.2109
7. Clinical trial for benefit of others	77	33 (42.9)	25 (32.5)	19 (24.7)	0.1464
8. Participate in trials even if more time consuming or additional tests	75	30 (40)	20 (26.7)	25 (33.3)	0.3654
9. Privacy protection by the university	74	42 (56.8)	19 (25.7)	13 (17.6)	<0.0001
10. Safety by University sponsored trial	74	38 (51.4)	19 (25.7)	17 (23)	0.0059
